# Past Adversity Influencing Now (PAIN): perspectives on the impact of temporal language on the persistence of pain

**DOI:** 10.3389/fpain.2023.1244390

**Published:** 2023-09-18

**Authors:** Matt Hudson, Mark I. Johnson

**Affiliations:** ^1^ Centre for Pain Research, School of Health, Leeds Beckett University, Leeds, United Kingdom; ^2^ Mind Help Limited, Durham, United Kingdom

**Keywords:** pain, persistent pain, linguistic relativity, temporal language, linguistics, emotional memory image (EMI)

## Abstract

Persistent pain is a significant healthcare issue, often unresponsive to traditional treatments. We argue for incorporating non-biomedical perspectives in understanding pain, promoting more comprehensive solutions. This article explores how language, specifically time-related terms, may affect the persistence (stickiness) of pain. We delve into how language influences one's experience of the world, especially in understanding pain through spatial metaphors. Notably, time perceptions differ across languages and cultures and there is no absolute construct of temporal pain experience. In English, time is viewed linearly as past, present, and future. We introduce a framework called Past Adversity Influencing Now (PAIN) which includes various temporal phases of pain; Past Perfect, Past Imperfect, Present, Future Imperfect, and Future Perfect. We suggest that past negative memories (emotional memory images) can “trap” individuals in a “sticky” pain state. We speculate that the process of diagnosing pain as “chronic” may solidify this “stickiness”, drawing from the ancient Greek idea of “logos”, where pain communicates a message across time and space needing recognition. Our PAIN framework encourages examining pain through a temporal lens, guiding individuals towards a more positive future.

## Introduction

In this article, we explore the influence of temporal (time-based) language on the persistence (stickiness) of pain (1). Although the study of language and pain is not new, we hope to add a novel perspective by appraising the temporality of pain language through the lens of linguistic relativity (2–4), i.e., how language shapes a person's lived experience. We argue that the Sapir-Whorf hypothesis that a person's perception and construction of experience is determined by the structure of their native language and culture, is of critical relevance to the subjectivity of a person's pain (5–7). The words used to represent time, place, space and experience are intricate representations of complex systems of language, and therefore a person's use of the word “pain” may not necessarily resemble their actual experience of pain (8). We model pain experience onto a novel framework termed Past Adversity Influencing Now (PAIN) to consider how temporal language may promote pain persistence by trapping a person within the health practitioner's time frame of recovery, not their own. We explain how the notion of Past Adversity Influencing Now (PAIN) comprising a Past Perfect, Past Imperfect, Present (now), Future Imperfect and Future Perfect may assist pain practitioners in reconfiguring temporal language to accelerate healing and recovery.

## Temporality and mental models of reality

Time is a fundamental human experience and a construct of conceptual thinking. Philosophically, debates exist between presentism (only the present moment exists) and eternalism (past, present, and future coexist) (9). In the theory of general relativity, time refers to a dimension intertwined with space. In biology, time is a variable associated with growth, ageing, and circadian rhythms. In psychology, time is considered a perceptual experience of duration, order, and intervals between events, resulting in a subjective experience of “the passage of time”. Thus, time may be considered a physical thing (objective) and/or a psychological construct (subjective), and malleable in both instances.

### Temporality and language

Language, using linguistic symbols such as words, enables humans to communicate information about abstract thoughts and ideas, and objects and events in the external environment (8). A person's “lifeworld” denotes a person's subjective construction of reality formed within their life circumstances (10), and metaphoric language appears to be critical in shaping perceptions of reality and subjective experience (2–4). People who speak different languages attend to and encode different aspects of the world and think and perceive different features from similar situations; this affects how a person constructs and experiences their reality within the conditions and circumstances of the situation (11, 12). Thus, a person's lifeworld may be malleable through the reconfiguration of their language narrative.

Humans learn a vocabulary of time (temporal language) during physiological development. In English, temporal language maps into the nature of experience, including pain, using a *horizontal* framework as past, present and future. However, people from different linguistic backgrounds conceptualise time using different domains, e.g., horizontal-vertical, left-right, front-back, East-West, distance-quantity, stationary-moving, and limited-open ended (12, 13).

In English language, linguistic constructions for time and space overlap, using metaphors that locate events on a horizontal mental timeline, i.e., front-back metaphors of the future being in front (“looking forward”) and the past being behind (“looking back”) (14). However, in the Andes language of Aymara, future events are framed as behind and past events in front (15). In Mandarin, time and order may be described using a vertical metaphorical construct of up and down, e.g., shàng (up) instead of last and xià (down) instead of next (11, 16). For English speakers, who write from left to right mental timelines are represented on a left (before/past)-right (after/future) axis, but this is reversed for languages writing right to left, e.g., Arabic and Hebrew. Thus, time is represented in different ways in different languages and in accordance with common spatial metaphors used in the respective language (17). Health practitioners should be mindful of language (conceptual) and cultural diversity when discussing the time course of pain with non-English speakers. In the remainder of this article, we will focus on temporality from the perspective of the English language.

### Temporality of pain experience

The intersection between time and pain is typically experienced in a relational context, which is to say, in relation to something else. This might involve contrasting the intensity or quality of pain across different points in time, or it may involve comparing pain within a specific duration but in correlation to another factor. A person's lifeworld is in continuous flow, whereby each thought influences the next moment. Adams describes “timescapes” as a lens through which humans understand their lifeworld, and temporal relations with the world can rupture when a person's relationship with themselves or others in their world changes, for example, through episodes of pain associated with physical trauma (18, 19). When this happens sense making needs to be rebuilt to differentiate cause from effect and this offers future directions that are plausible based on an understanding of the past and present (18, 19). From this perspective, a person's experience of pain can be shaped not only by their personal temporal understanding but also through the interaction with the temporal experiences of others within the same culture (20). This suggests that changes in the language relating to time and pain might have effects that reach beyond the individual, potentially impacting a group-level experience. This is due to the nature of pain, which is inherently social and relational. Thus, the influence of shared temporal experiences must be considered alongside individual factors in understanding and managing pain (21).

Temporal language is the process of time-framing events in sentences using transitional words and phrases that indicate the order, direction, and flow of ideas, meaning, context or events. Transitional words and phrases that function to define, limit, and restrict time (temporal connectives) tell the listener or reader *when* something (an action) is happening and enable the meaning and context of information to flow. Common examples include first, second, now, then, before, after, later, eventually, finally, to begin with, in a moment, and suddenly.

Grammar is the system and structure of a language. In traditional English grammar, tenses are used to reference time, i.e., a tense is the arrangement of a verb that enables the expression of time. Verb tenses describe something happening now (present), had happened (past), or will happen (future) and comprise the following forms (aspects): Simple, Continuous (Progressive), Perfect, Perfect Continuous (Perfect Progressive). A perfect tense refers to completed actions or states, and a continuous (imperfect) tense describes incomplete actions or states that are continuous or repeated (e.g., “was doing”). Thus, twelve basic English tenses arise:

• Present Simple, Present Continuous, Present Perfect, Present Perfect Continuous
• Past Simple, Past Continuous, Past Perfect, Past Perfect Continuous
• Future Simple, Future Continuous, Future Perfect, Future Perfect Continuous

Perfect (completed) and imperfect (continuous) aspects of tenses offer supplementary steps within a person's structure of time and help to chart a person's pain history. Past Perfect tenses refer to past actions or states that were completed (resolved) before another action started. Past Continuous tenses refer to past actions or states that were ongoing (unresolved) before another action started. Thus:

• Past Simple: Describes an activity that started in the past—“I was in pain”.
• Past Continuous: Describes an unfinished (ongoing) activity in the past—“The pain was hurting when … [I saw a doctor]”
• Past Perfect: Describes an action that was completed in the past—“I had pain in 2021 before… [I saw a doctor]” or “I had never been in pain before … [I saw a doctor]” or “I only recovered because … [I saw a doctor]”
• Past Perfect Continuous: Describes an action that started in the past and continued until another time in the past—“I had been hurting until [I saw a doctor]”, “I wanted to see a doctor because I had been hurting all day at work”, and “How long had you been hurting before … [you saw the doctor?]”

The Past Perfect Continuous tense is a useful way to suggest cause and effect.

The Past *Imperfect* tense describes an unfinished action and is also known as the past continuous or past progressive. In the context of this article, and from a perspective of utility, *imperfect* is a word that not only conveys unfinished actions but also imperfect “situations” that may influence the present (now), including thoughts about the future.

Mapping pain onto a simplified temporal framework of perfect and imperfect tenses reveals the relationship between unfinished (ongoing) situations from the past (i.e., adverse events), and bodily pain, including thoughts about the future, which can only exist in the present (i.e., now).

When applied as a verb “pain” necessitates a detachment of a person's identity (not necessarily from their physical body, but more so from their ego). For example, “The wound pained me.” inherently creates a distinction between the individual and the wound, with the wound becoming an object that can be acted upon through an external attribution to the wound causing the discomfort. This contrasts with phrases like “My pain makes me suffer”. where the pain is internalised and objectified and becomes part of one's perception of self, the personal “my/me/ego”. In Buddhism, pain is seen as the fuel for transcendence and thus the ego is let go (22).

### Nominalisation of pain experience

In linguistics, “nominalisation” is the process of converting verbs, adjectives, or other word types into nouns. Nouns are crucial as they name or identify entities or ideas, shaping our understanding of the world. By using universally understood nouns, we foster shared comprehension. Nominalisation allows us to assign existence or identity to actions, qualities, and concepts, differentiating them from others.

The nominalisation of pain generally goes unnoticed. In everyday conversation, the word pain is used to convey an experience of an inner state of the body that lacks distinctiveness [for debates on the *nature* of pain see (23–27)]. The common viewpoint, that pain is representational of something in the world, e.g., bodily adversity, harm, tissue damage etc., is contested by an alternative viewpoint, less widely accepted, that pain is a free-floating sensation, and not *about* [representational of] anything [for review see (23)].

**Figure 1:**
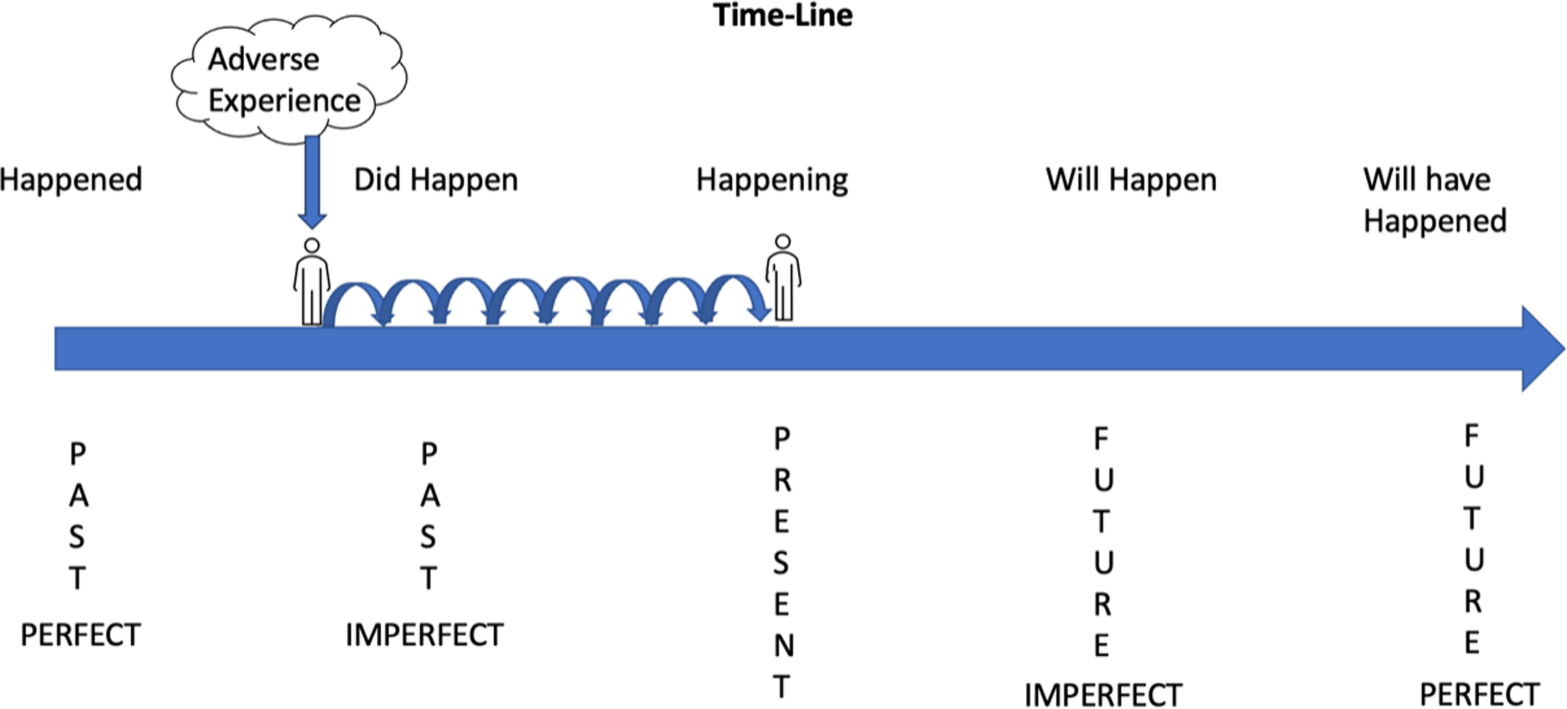
Pain experience and temporal language. Past imperfect tenses set the context of what *was* happening while another event occurred. For example:
•“I was studying when a stabbing pain started in my leg”. (ongoing action of studying)•“I was playing rugby when I noticed my arm hurting”. (ongoing action of playing rugby)Past imperfect tenses also describe habitual actions or states such as repeated actions or ongoing situations. For example:
•“Every day, I was complaining of pain”. (habitual action of complaining)•“She was always talking about her pain”. (habitual action of talking about pain)Past adverse experiences influence thoughts, expectations and predictions of the “now” (present) resulting in stickiness of pain expressed via future tenses:
•Future Imperfect Simple—“I will hurt tomorrow”.•Future Imperfect Continuous—“I will be hurting tomorrow”•Future Imperfect—“I will have no pain tomorrow”.•Future Perfect Continuous “On my next birthday I will have been hurting for 7 years”.If a person is in the present and says “I will have no pain tomorrow” their language is Future Imperfect because they are remaining in the present to look forward at an action that is incomplete. If a person is in the present and says “I can't wait until my next birthday to look back on how my pain cleared” their language is Future Perfect because they have gone past the incomplete action to a point in the future to look back at the completed action. Hence, “By then I will have had” is constructive language because it completes an imperfect or incomplete action and creates a perfect future seen as the problem has been resolved.

Pain is a subjective experience *and* the topic (object) of that experience. Biomedical science investigates pain via a materialistic and reductionist paradigm that uses equipment to detect a *concrete* (physical) thing, e.g., chemical, or neural substrates as direct or indirect markers (signatures) of pain. Cohen et al. argue that pain is not a “thing” (28) and Bourke argues that pain is “a type of event” (29). Even when arguing that pain is not a thing, pain becomes nominalised! The examples provided in the caption of Figure 1 reveal the nominalisation of pain, i.e., the English language allows a person *to hurt* (verb) but not *to pain*. In this instance pain is not expressed as a verb. In the English language, pain has become a noun, representative of a “thing”, and we contend that this may influence the stickiness of pain. There is, however, the use of “pained” where pain is used as a transitive verb, although this is seldom used in modern-day speech. In the English language, nominalisation of pain has the potential to create time-related dimensions of pain that convey fallacies, misnomers and pain narratives that are more insidious than depicted in the caption for Figure 1. For example, “my diagnosis is chronic pain” may inadvertently shape a person's belief of no hope for recovery, when this might not be the case, fostering a pessimistic view of future health.

By reflecting on the nominalisation of pain and temporal markers of pain experience, opportunities arise for scholars, patients, and practitioners to participate together in a process of discovery of how Past Adversity Influences Now (PAIN). For example, we postulate that, in some people, nominalisation “freezes” a person's living experience of pain in the present, thus “blocking the flow” of a person's reality. This “locks” pain in a lived experience of the past and in so doing collapses future possibilities into a reality that retains the adversity of an imperfect past. This stagnant state of mind and body may create worry, rumination and catastrophising, a significant psychological factor related to the persistence of pain and disability (30–33). The interplay of biological, social, and psychological disturbances that contribute to chronic pain unresponsive to treatment has the nickname “stickiness” (1, 34).

## Past Adversity Influencing Now (PAIN)

A person experiences sensations and emotions, including pain, only in the current moment. While pain is experienced in the present, it is influenced by past events and potential future occurrences. Pain emerges from the integration of sensory, emotional, and cognitive elements of present moment physiological processes, along with memories of past experiences, both conscious and unconscious. A discussion of the nature and formation of memories is beyond the scope of this article; suffice to say that the conventional synaptic and bioplastic model of memory (35) has limitations and has been challenged (36).

When discussing a person's pain, practitioner and patient are often unaware of the power that time-based narrative brings to bear on their respective realities of experiences and situations. Health practitioners and their patients discuss pain within a temporal construct of the past, “What happened to cause pain”, present “How does pain affect you now”, and future “What should be done to effect recovery”. This time-based narrative creates a sequential construct whereby pain experienced in the “now” (Present Pain), matches expectations with what happened in the past (Past Perfect), and a prediction that pain will resolve (Future Perfect—positive prognosis). For example, a transient inconsequential pain now (Present Pain) described as “I had stubbed my toe” (Past Perfect), is expected to disappear within seconds “Ouch! the pain will disappear in a moment” (Future Perfect), and it usually does. Likewise, a person reporting that the intensity of their pain is decreasing with medication and no longer interferes with activities of daily living (Present Pain) due to an accident two weeks ago (Past Perfect) will expect their pain to disappear in a few weeks more (Future Perfect).

### An imperfect past contributing to an imperfect present and future

Imagine pain persists beyond the expected duration of healing; “An accident happened a long time ago, yet I'm still in pain despite the medication”. Temporal language may become structured as “I was arguing with my partner when the accident happened, and I am still in pain today” (Past Imperfect). When pain remains unresolved, despite treatment, a temporal narrative of “I have been in pain (hurting) for nearly two years, and I will just have to learn to live with this pain” (Future Imperfect) emerges. The person's temporal language is simple, easy to follow and leads to a logical conclusion. Based on the experience of living a long time with ongoing (unresolved) pain (Past Imperfect) the patient expects that pain (Present Pain) will always be there (Future Imperfect), i.e., the person's pain becomes “sticky”.

## Emotional Memory Images (EMIs) and PAIN

**Figure 2:**
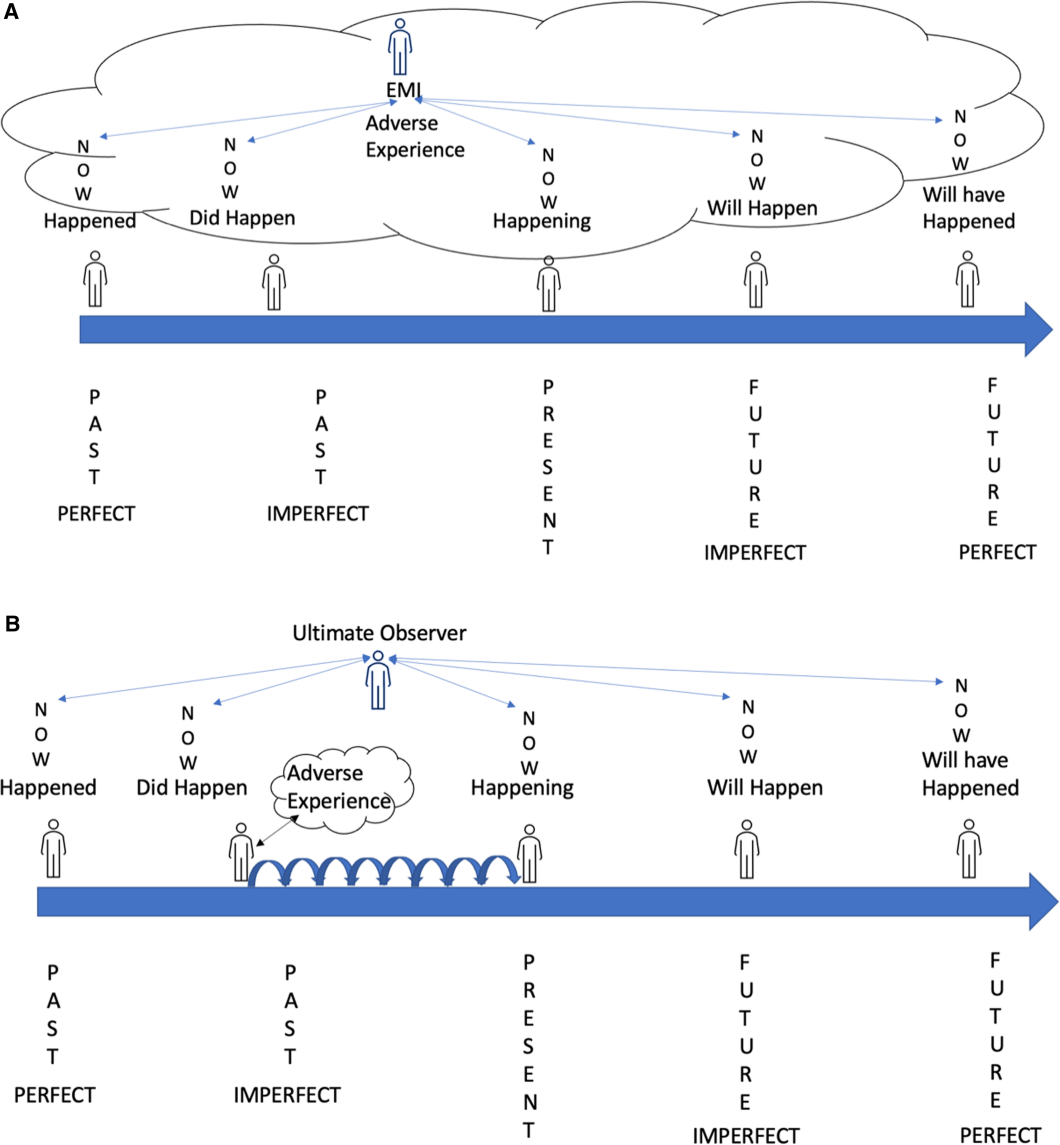
(**A**) Emotional memory images and temporal language. (**B**) Curious exploration of the temporal positions of pain experience.

In 2021, we proposed a model of psychophysiological “dis-ease” whereby stress responses from first-time, novel, and unprecedented traumatic emotional experiences are rapidly learnt and then retriggered later in daily life when a person encounters a reminder of the original traumatic experience (37). Central to our proposal was the concept of Emotional Memory Images (EMIs) coupled to the hypothalamic-pituitary-adrenal (HPA) axis and stress like responses, e.g., flight, fight, freeze, tonic immobility, and quiescent immobility (38, 39). We defined EMIs as “Trauma induced, non-conscious, contiguously formed multimodal mental imagery, which triggers an amnesic, anachronistic, stress response within a split-second”. (39). We argued that EMIs are re-triggered by encounters broadly akin to the original experience, continually revivifying the past and contributing to states of psychophysiological dis-ease, influencing the persistence of pain (40–42). Importantly, the anachronistic nonconscious nature of EMIs renders the person amnesic to the original traumatic experience and bereft of reasons why they experience persistence of pain (Figure 2A). Thus, clearing (unlearning) EMIs may alleviate, at least in part, autonomic stress-like responses associated with past adversity, thereby reducing allostatic load (43).

We described a therapeutic approach, Split-Second Unlearning (37), whereby practitioners screen for micro-expression(s) signifying an in-the-moment stress response representative of the presence of an EMI. The practitioner makes the patient aware of these micro-expressions and encourages curious exploration of the influence of EMIs on temporal positions of their condition (pain experience) so that the patient can learn to separate the EMI from the stress, clearing the EMI, metaphorically or literally, and alleviating discomfort (Figure 2B). For case vignettes see (37, 39, 40).

## Curiously moving time to positively impact PAIN

There is a wealth of evidence that time and pain are intrinsically linked (44). Bodily pain slows down the perception of time (45) and the temporal state of mind shapes pain experience for better (placebo) or worse (nocebo) (46, 47). Therapeutic interventions such as cognitive behavioural therapy (CBT) mindfulness and eye movement desensitisation and reprocessing (EMDR) have temporal components within the methodology designed to positively influence the relationship between time and pain (48–51). Indeed the core tenet of mindfulness is to focus one's attention on the present moment (52). The psychological aspect of pain is driven by the perception of what was, what is and what shall be, and we contend that EMIs act as placeholders bringing past pain into the present and advocate approaches that address nonconscious motivational traits detrimental to recovery.

Our Split-Second Unlearning model of psychophysiological dis-ease (37) was developed from the principles and practice of Neurolinguistic Programming (NLP). The field of NLP describes nonconscious motivational traits called metaprograms that show specifically how much time is needed before an individual will decide on something, such as positive change in attitude and behaviour (53). Strategies used by people to decide on something are:

• Automatic—a person is immediately convinced of an idea/situation.
• Number of times—It usually takes a person a few goes before they are convinced (e.g., “Third time lucky”).
• Period of time—It can take a month or more for a person to be convinced as they need time to pass before they can accept or decide something.
• Consistent—No matter how much time a person is given they are still not convinced.

Temporal language that aligns with a patient's decision strategy can be used as a motivational tool to influence health outcomes (54). Examples include,

• “You’ll be up and about in no time at all”—Automatic
• “It may take 2 or 3 days of exercise before you begin to notice how much better you are feeling”—Number of times
• “You're going to need some time before you start feeling any improvement … [the practitioner pauses for 1 min] … and now that you’ve had time what are you beginning to notice?”—Period of time
• “The thing is Mr Brown even when you are back walking 15 miles a day you will still have doubts about whether the pain will stay away for good!”—Consistent

Self-reports of pain include a level of nonconscious bias (55). For example, a person's measure of time can become distorted as they judge their experience as being longer than it actually was (56, 57). Pain assessment tools inherently focus on past and present pain. The McGill Pain Questionnaire (MPQ) asks patients to rate their pain “felt during the past week”, and this may inadvertently reinforce and even intensify pain by reviving dormant memories (58, 59). Asking patients to complete pain body maps, denoting pain as a static experience may also contribute to stickiness. Moreover, self-reports may fall foul of the “peak-end rule”, a psychological heuristic (mental shortcut) to quickly solve problems and make judgements (60), whereby people report pain from the peak (most intense) and the end (most recent) of their pain experience (61).

Types of cognitive heuristics that draw on the past when reporting pain experience may include:

1. Availability Heuristic: Judgments based on the most *recent* episode of pain.
2. Anchoring Heuristic: Judgements based on the *initial* episode of pain, when pain first appeared.
3. Representativeness Heuristic: Judgements based on the similarity of pain to *previous* typical pain experiences.
4. Familiarity Heuristic: Judgements based on *previous* pain behaviour that was successful under similar circumstances, such as gaining access to pain medication during a clinical consultation.

These heuristics not only shape patient expectations and coping strategies, but also influence healthcare providers’ communication and treatment plans (62–64) [see also (65) for a greater insight into the temporal aspect of pain]. Greater awareness of how these unconscious and conscious considerations of past experience influence a person's experience of the present (“now”) can be used in clinical practice to curiously move time to positively impact PAIN.

## Careless use of temporal language in clinical practice

There is increasing awareness of the need to use positive and constructive pain language (66), yet less attention has been given to the insidious nature of *temporal* pain language that may be detrimental to patient outcomes. In our article, for example, we default to conventional pain nomenclature that suggests permanence, such as “persistent pain”, “chronic pain”, and “intractable pain”, potentially leading patients to feelings of anxiety, depression, and fear-avoidance of activities that may intensify pain, resulting in physical and emotional deterioration. Moreover, “persistent”, “chronic”, and “intractable” may skew the attitudes of healthcare professionals towards symptom management rather than broader causes that may assist recovery, and influence language used in clinical consultation. Examples include,

• “You will have to learn to live with it”
• “There's nothing more we can do”
• “You’ll be on this medication for life”
• “You will have to learn to pace yourself”.

These examples show how the practitioner condemns the patient to a reality of pain and suffering that did not exist before the practitioner spoke. However, the statements do not hold logically as there is no knowing what the future holds.

An awareness of the danger of careless use of temporal language can be traced back to ancient Greek philosophers such as Aristotle and Plato. Both Aristotle and Plato discussed the concept of “logos” (67, 68). For Aristotle “logos” was one of three persuasive modes, alongside “ethos” and “pathos”. It denotes logical appeal in persuasion. Both Aristotle and Plato emphasised its importance but with varying interpretations. For Aristotle, it was a principle in human thought and nature. An example: in diagnosing “sticky pain”, while it appears illogical, practitioners aim for a logical explanation. Plato viewed “logos” as a cosmic truth, whereby pain is a message awaiting acknowledgement. Here pain is considered more of an emotional than sensory experience, that belonged in the soul. If pain stickiness is driven, at least in part by emotional memories as proposed in our framework of PAIN and Split-Second Unlearning theory, then the logos of both philosophers will stand, switching focus from a mechanistic biomedical model of pain in the brain to a model of pain that encompasses a metaphysical mind.

It is possible to learn new ways to talk about time by learning new metaphors and in doing so it is possible to reconfigure space-time associations and non-linguistic representations of time (69). We encourage practitioners to experiment with temporal metaphors that embrace the logos of Plato and Aristotle to help the person break free from the confines of a mechanistic biomedical model. That is, to explore time-based metaphysical metaphors for a metaphysical mind rather than a mechanistic brain. For example:

• “When the mind is willing healing can happen very quickly”
• “You will know when the time is right for you, to begin again”
• “Some patients get this and transform their lives immediately, some take a few days longer, and others can take up to a month or so before they really begin to feel the benefits, there are even those who feel the benefits and will never admit to it coming from this work and that's okay, too”.

The final statement utilises all the temporal decision-making metaprograms and may be used to address all patients.

## Conclusion

Early life adversities negatively affect health and increase the risk of an episode of pain persisting (70). We contend that adverse experiences may trap individuals in their perception of time, making pain “sticky”, and describe this phenomenon as a “Past Imperfect” when one's past negatively impacts their present outlook and future expectations. Linguistic studies reveal that English speakers represent pain in a temporal manner, specifically in a horizontal space. We have conceptualised this as “Past Adversity Influencing Now” (PAIN), suggesting that prior negative experiences can keep individuals trapped in a specific time perception, which affects their pain experience. Different languages and cultures have varied mental models of time, and existing representations can change, providing a path to healing. Contemporary views in pain management suggest that conventional pain treatments might not always benefit patients. Instead, exploring the linguistic aspects of pain might offer more holistic healing. Health professionals are encouraged to use language as a tool to help patients explore their pain experiences. Interdisciplinary research, combining linguistics, psychology, and medical science, is essential for a comprehensive understanding of pain.

## Data Availability

The original contributions presented in the study are included in the article/Supplementary Material, further inquiries can be directed to the corresponding author.
